# c-di-GMP is required for swarming in *E. coli*, producing colanic acid that acts as surfactant

**DOI:** 10.1128/mbio.00916-25

**Published:** 2025-05-06

**Authors:** YuneSahng Hwang, Marta Perez, Rebecca Holzel, Rasika M. Harshey

**Affiliations:** 1 Department of Molecular Biosciences and LaMontagne Center for Infectious Diseases, The University of Texas 196204, Austin, Texas, USA; University of Washington School of Medicine, Seattle, Washington, USA

**Keywords:** cyclic-di-GMP, swarming, colanic acid, DgcO, DgcM

## Abstract

Many bacteria use flagella to swim individually through bulk liquid or swarm collectively over a semi-solid surface. In *Escherichia coli*, c-di-GMP inhibits swimming via the effector protein YcgR. We show in this study that, contrary to its effect on swimming, a certain threshold level of c-di-GMP is required for swarming. Gene expression profiles first indicated that several c-di-GMP synthases*—dgcJ*, *dgcM*, and *dgcO*—were upregulated during swarming. Of these, we found DgcO to play a critical role in promoting the production of colanic acid—one of the three major exopolysaccharides in *E. coli*. DgcO has been reported to increase poly-β-1,6-N-acetylglucosamine (PGA) synthesis in *E. coli* as well. We show that colanic acid has hitherto-unknown surfactant properties that are expected to aid swarming.

**IMPORTANCE:**

It is well established that, in bacteria, c-di-GMP inhibits flagella-driven motility at various points in the pathway. Concomitantly, elevated c-di-GMP levels induce the expression and synthesis of a variety of exopolysaccharides that enmesh the bacteria in a biofilm, thereby also interfering with the flagella function. This study reports the surprising finding that, in *Escherichia coli*, the exopolysaccharide colanic acid is required to enable surface navigation and that the diguanylate cyclase DgcO is employed for this purpose. For surface navigation, there appears to be a sweet spot where c-di-GMP levels are just right to produce polysaccharides that can serve as surfactants and wetting agents rather than promote the formation of biofilms.

## INTRODUCTION

Cyclic-di-GMP (c-di-GMP) signaling offers bacteria lifestyle choices, the most prevalent being a choice between moving freely or settling in a biofilm (1–3). *Escherichia coli* has multiple diguanylate cyclases (DGCs) and phosphodiesterases (PDEs) that synthesize and degrade c-di-GMP, respectively (4). In free-swimming or planktonic *E. coli*, DgcN, DgcO, DgcQ, and DgcE are reported to be some of the most active DGCs, and PdeH is the most active PDE (5–7). Elevated c-di-GMP levels resulting from the inactivation of PdeH inhibit chemotaxis and swimming speed but have only a moderate effect on swarming (8). The inhibitory effect of c-di-GMP on swimming motility is well-studied and is orchestrated via interaction of the effector protein YcgR with both the flagellar motor and the rotor (5, 8–13). High levels of c-di-GMP promote biofilm formation (1, 3, 14). *E. coli* biofilms consist of various extracellular polymers and polysaccharides (15), including colanic acid (CA), cellulose, curli fimbriae, and poly-β-1,6-N-acetyl-d-glucosamine (PGA) (2).

Swarming presents unique challenges not encountered while swimming (16, 17). These include limited water availability necessary for flagella to work and formidable physical forces such as surface tension, friction, capillary, and viscous forces. Bacteria overcome these challenges by secreting surfactants to reduce surface tension and osmolytes/wetting agents to draw water to the surface, altering cell shape to improve side-by-side alignment to lower viscous drag, and increasing flagella numbers or recruiting special stator-associated proteins to enhance motor power (17–20). In the laboratory, swarming is observed on media solidified with agar, whose consistency can range from semi-solid to solid depending on the agar concentration (0.5–0.7% or 1–2%, wt/vol), presenting differing levels of navigational challenge. Bacteria that only swarm on the lower range of agar concentration have been classified as “temperate,” and those that swarm on the higher range have been classified as “robust” swarmers (17). *E. coli* and *Salmonella* swarmers belong to the former category, while *Proteus mirabilis* and *Vibrio parahaemolyticus* fall under the robust category; the latter set of bacteria increases flagella production to enhance motor power. Some temperate swarmers such as *Serratia marcescens* and *Bacillus subtilis* secrete copious amounts of surfactants and/or increase flagella numbers (19).

*E. coli* and *Salmonella* neither increase flagella numbers nor are reported to secrete specific surfactants (21–24). These bacteria require nutrient-rich media for swarming, with *E. coli* being particularly fastidious, needing in addition 0.5% glucose and a specific type of agar (Eiken agar) (24, 25). Several studies suggested that lipopolysaccharide (LPS) might serve to attract water to the surface in these bacteria and implicated an additional role for flagella rotation in enabling a wet surface (26–29). One of these studies identified non-swarming mutants in the LPS biosynthetic pathway of *Salmonella* and suggested that the hydrophilic nature of LPS may serve as a wetting agent (26), while another detected high osmotic pressure at the leading edge of an *E. coli* swarm using fluorescent liposomes and suggested that a secreted high molecular weight substance (perhaps LPS) extracts water from the underlying agar to enable motility (27). Microarray data showed that LPS synthesis was upregulated specifically in *Salmonella* swarms (30). Non-swarming mutants of *Salmonella* also mapped to the CA biosynthesis pathway (26). CA is a polyanionic heteropolysaccharide composed of repeating hexo-saccharides and could also serve as a potential secreted wetting agent. We note that, in the surfactant literature, the terms “surfactant” and “wetting agent” have been used interchangeably (17, 31); here, we make a distinction and use surfactant to mean “lowering of surface tension” and wetting agent to mean “ability to attract water.”

The present study was initiated to understand the significance of the differential expression of several DGCs and PDEs observed in RNAseq data collected during *E. coli* swarming. Of the upregulated DGCs, DgcJ is reported to associate with and activate NfrB glycosyl transferase to produce an exopolysaccharide that is a receptor for phage N4 (32, 33), DgcM is reported to regulate the production of curli fibers through the *csgABC* operon (34–36), and DgcO is recognized for its ability to bind oxygen via its heme domain (37) and to enhance PGA synthesis by transcriptional mechanisms (38); PGA synthesis is also regulated by post-transcriptional mechanisms (38, 39). Deletion analysis of the upregulated genes indicated that only the absence of DgcM and DgcO had a negative effect on swarming, the absence of DgcO being more critical. We therefore investigated the role of DgcO and found that its swarming defect is caused primarily by a lack of CA production. We demonstrate that CA has surfactant properties that, along with its expected wetting properties, assist swarming.

## RESULTS

### DgcO plays a critical role in swarming

**Fig 1:**
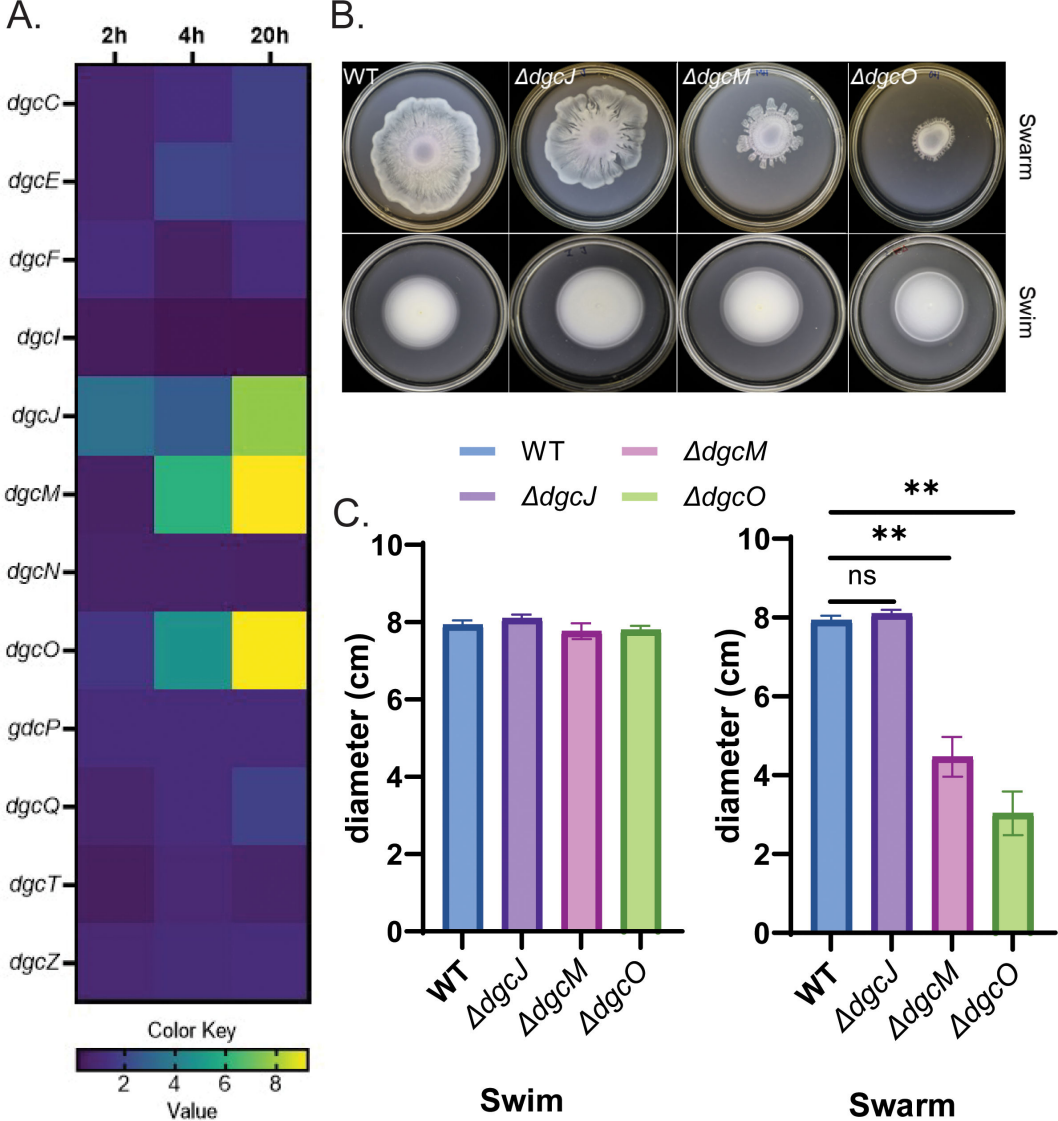
Identification of DGCs that contribute to swarming motility. (**A**) Comparison of fold changes in gene expression of WT *E. coli* DGCs during the time course of swarming. RNAseq data collected at 2, 4, and 20 h were normalized to those from planktonic cultures (*n* = 4). (**B**) Comparison of swimming (0.3% agar) and swarming (0.5% agar) motilities of indicated DGC deletion mutants in the WT strain. Plates were dried for 1 h under the hood and incubated at 30°C for 18 h (*n* = 3). (**C**) Plot of diameters across the zones of bacterial swimming or swarming shown in panel B. Calculated *P* values are indicated: *, <0.05, **, <0.01, or ***, <0.0001. NS, not statistically significant.

Mutations in *dgcJ* were recovered during a screen for motile antibiotic-resistant mutants emerging from the edge of an *E. coli* swarm, suggesting that DgcJ is active in the swarm (40). That c-di-GMP enzymes might be differentially regulated during swimming vs swarming was inferred by the differential effect of the absence of PdeH on the two types of motilities (8) (Fig. S1A). The estimation of c-di-GMP levels in WT under the two conditions using a riboswitch-based c-di-GMP sensor (41) showed these to be similar (Table 1). However, there was a much larger increase of c-di-GMP in the Δ*pdeH* mutant in swim compared to swarm cells (Fig. S1B and Table 1). These observations motivated us to examine RNAseq data (generated as part of a different project; see Materials and Methods) for expression of DGCs and PDEs during a 2–20 h time course of swarming. Compared to planktonic or swim cells, we observed that the expression of *dgcJ*, *dgcM*, and* dgcO* was elevated approximately threefold to eightfold during this time course (Fig. 1A). Among the PDEs, *pdeH* was drastically downregulated during swarming, with a reduction of over 100-fold in the measured raw values (Fig. S1C), while *pdeO* and *pdeR* were significantly upregulated (Fig. S1C). The contribution of the upregulated DGCs and PDEs to both swimming and swarming was investigated next by deleting them individually (Fig. 1B and C and Fig. S1D). Deletion of neither *pdeO* nor *pdeR* had any effect on either motility (Fig. S1D). Deletion of *dgcJ* also had no observable effect, deletion of *dgcM* decreased swarming, while deletion of *dgcO* had a consistently worse outcome for swarming (Fig. 1C). Under standard conditions for preparing swarm plates, we dry freshly poured plates for 1 h in a hood under laminar flow. When plates were dried for suboptimal periods, where *dgcM* and *dgcO* mutants had better outcomes, the double Δ*dgcMO* strain had a pronounced swarm defect (Fig. S2), suggesting that *dgcM* and *dgcO* likely jointly contribute to stimulating some downstream pathway(s). Δ*dgcO* cells observed under the microscope at 4 h, a time point when active motion is observable in WT, were seen to be moving as well as WT (Movies S1 and S2), showing that the defect was not in motility but in their ability to advance across the surface.

**TABLE 1:** c-di-GMP levels estimated from a riboswitch-based sensor

Strain	Relative c-di-GMP level to WT	Condition
WT(MG1655)^*a*^	1 ± 0.008	Swim
Δ*pdeH*	2.88 ± 0.11	Swim
Δ*dgcO*	0.82 ± 0.03	Swim
WT + pPdeH (10 µM IPTG)	0.75 ± 0.04	Swim
Δ*dgcO* + pDgcO (0% ara)	1.28 ± 0.37	Swim
Δ*dgcO* + pDgcN (0% ara)	1.5 ± 0.09	Swim
Δ*dgcO* + pDgcN + (0.05% ara)	5 ± 0.56	Swim
WT(MG1655)	1 ± 0.093	Swarm
Δ*pdeH*	1.25 ± 0.031	Swarm
Δ*dgcM*	0.89 ± 0.0305	Swarm
Δ*dgcO*	0.57 ± 0.048	Swarm
WT + pPdeH (0 µM IPTG)	0.82 ± 0.111	Swarm
WT + pPdeH (10 µM IPTG)	0.67 ± 0.18	Swarm
WT + pPdeH (100 µM IPTG)	0.62 ± 0.08	Swarm
WT + pPdeH (1 mM IPTG)	0.21 ± 0.04	Swarm
Δ*dgcO* + pDgcO (0% ara)	0.70 ± 0.003	Swarm
Δ*dgcO* + pDgcN (0% ara)	1.48 ± 0.013	Swarm
Δ*dgcO* + pDgcO (0.05% ara)	2.56 ± 0.234	Swarm
Δ*dgcO* + pDgcN + (0.05% ara)	4.17 ± 0.400	Swarm

^*a*^ Estimates of c-di-GMP levels from WT cells taken directly from a swim and swarm plates (see Materials and Methods) were set to 1 for comparison with all other strains and conditions tested. Based on reported data for planktonic *E. coli* (41), these values are roughly equivalent to 8 pmol/mg protein. We are concerned here only with relative, not absolute, c-di-GMP values.

We conclude that both DgcM and DgcO play a positive role specifically during swarming, with DgcO being the dominant player. Going forward, we focused on the contribution of DgcO.

### c**-di-GMP is required for swarming**

**Fig 2:**
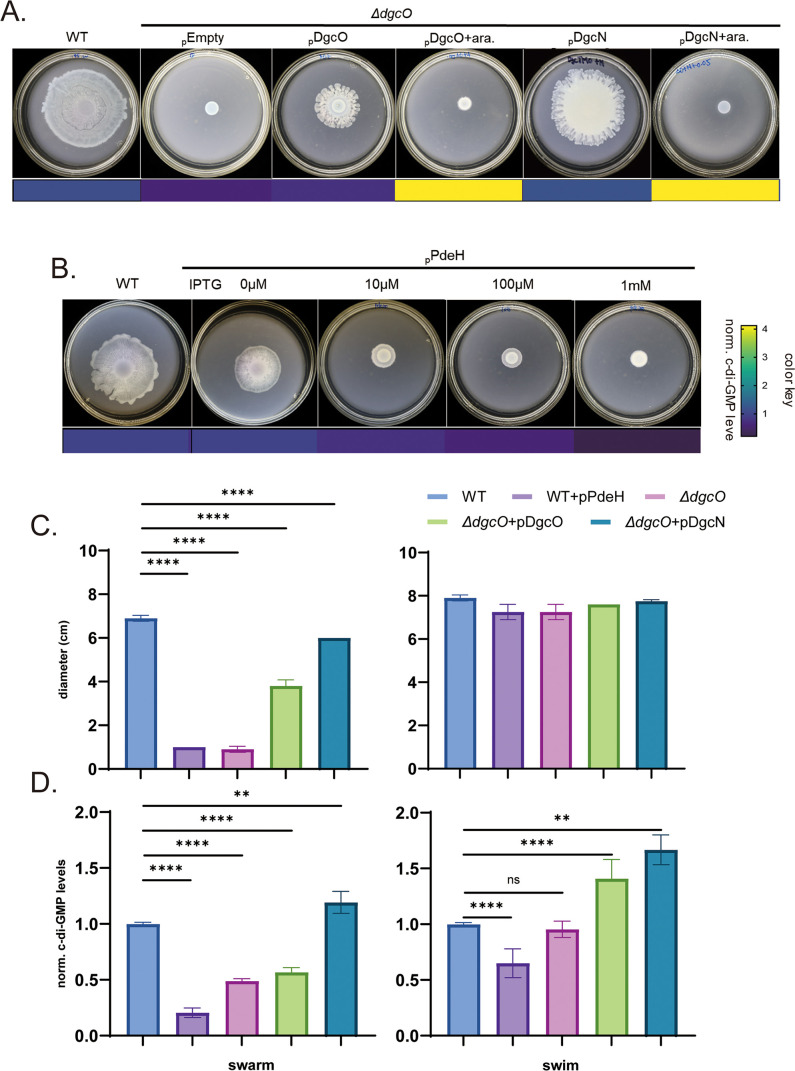
c-di-GMP is required for swarming. (**A**) Plasmids encoding *dgcO* (pDgcO), *dgcN* (pDgcN), or vector alone (pEmpty) under the control of pBAD promoter were introduced to the Δ*dgcO* strain, and swarming was compared to WT, with (+ara) or without addition of 0.05% arabinose (*n* = 4). The color key beneath the plates corresponds to relative c-di-GMP levels measured using the riboswitch-based c-di-GMP sensor, shown in panel B as heatmap (see Table 1). (**B**) A plasmid encoding *pdeH* (pPdeH) driven from the T5-*lacO* promoter was introduced into WT, and swarming was monitored at indicated IPTG concentrations (*n* = 2). c-di-GMP levels for all strains in this figure are found in Table 1, represented here by a heatmap beneath the plates. (**C**) Diameters of swim and swarm zones of strains indicated by a color key (*n* = 4); pDgcO and pDgcN data are without added inducer, and pPdeH with 10 mM IPTG. (**D**) Cells from swim/swarm plates in panel C were collected, and c-di-GMP levels were measured using the c-di-GMP biosensor. The values were normalized to the c-di-GMP levels in WT cells. Calculated *P* values are indicated: *, <0.05, **, <0.01, or ***, <0.0001. NS, not statistically significant. Color key as in panel C.

Complementation of Δ*dgcO* with a plasmid encoding *dgcO* driven from an arabinose-inducible promoter (pDgcO) partially rescued the swarming defect without added inducer (i.e., leaky expression; Fig. 2A); addition of arabinose inhibited rescue, suggesting that a narrow window of c-di-GMP levels contributed by DgcO supported swarming. To test whether the Δ*dgcO* phenotype is related solely to alterations in c-di-GMP levels, we introduced into the mutant a plasmid encoding *dgcN* (_p_DgcN). Leaky expression of *dgcN* was sufficient to rescue swarming, while addition of inducer was inhibitory (Fig. 2A and Table 1). The swarming inhibition observed by induction of pDgcN in the Δ*dgcO* mutant (Fig. 2A) was relieved by deletion of *ycgR*, showing that the inhibition at the higher levels of c-di-GMP is due to interference with flagellar function as established in studies on swimming (Fig. S3). To confirm the assessment that a certain threshold level of c-di-GMP is required for swarming, we took an opposite approach, reducing c-di-GMP levels in the WT by introducing a plasmid expressing *pdeH* (pPdeH) under the control of an IPTG-inducible promoter. Even leaky expression of *pdeH* had an inhibitory effect. Addition of a concentration as low as 10 µM IPTG reduced c-di-GMP levels below WT (Table 1), inhibiting swarming (Fig. 2B and C, left), but not swimming (Fig. 2C, right). c-di-GMP levels in all of these genetic backgrounds, in cells taken directly from the swarm and swim plates, are plotted in Fig. 2B and C and tabulated in Table 1. There was a correspondence between the measured c-di-GMP levels (Fig. 2D, left) and the extent of swarming (Fig. 2C, left), a correspondence that did not hold for swimming (compare panel C, right, with panel D, right), suggesting that, within the range tested, swarming is sensitive to c-di-GMP levels while swimming is not.

We conclude that, under our experimental conditions, c-di-GMP is necessary and supports optimal swarming within a narrow concentration range.

### Colanic acid is important for swarming

To investigate how c-di-GMP positively regulates swarming, we looked for suppressors that would restore swarming in a Δ*dgcO* strain and identified mutations in *fliT* and *fhuA. fliT* inhibits transcription of master flagellar regulator FlhDC (42, 43), and *fhuA* is involved in a TonB-dependent ferrichrome transport (44). The suppressors likely bypass the *dgcO* defect by other mechanisms, for example, by increasing flagella numbers in the *fliT* mutant (45) or lowering cellular iron levels (46).

**Fig 3:**
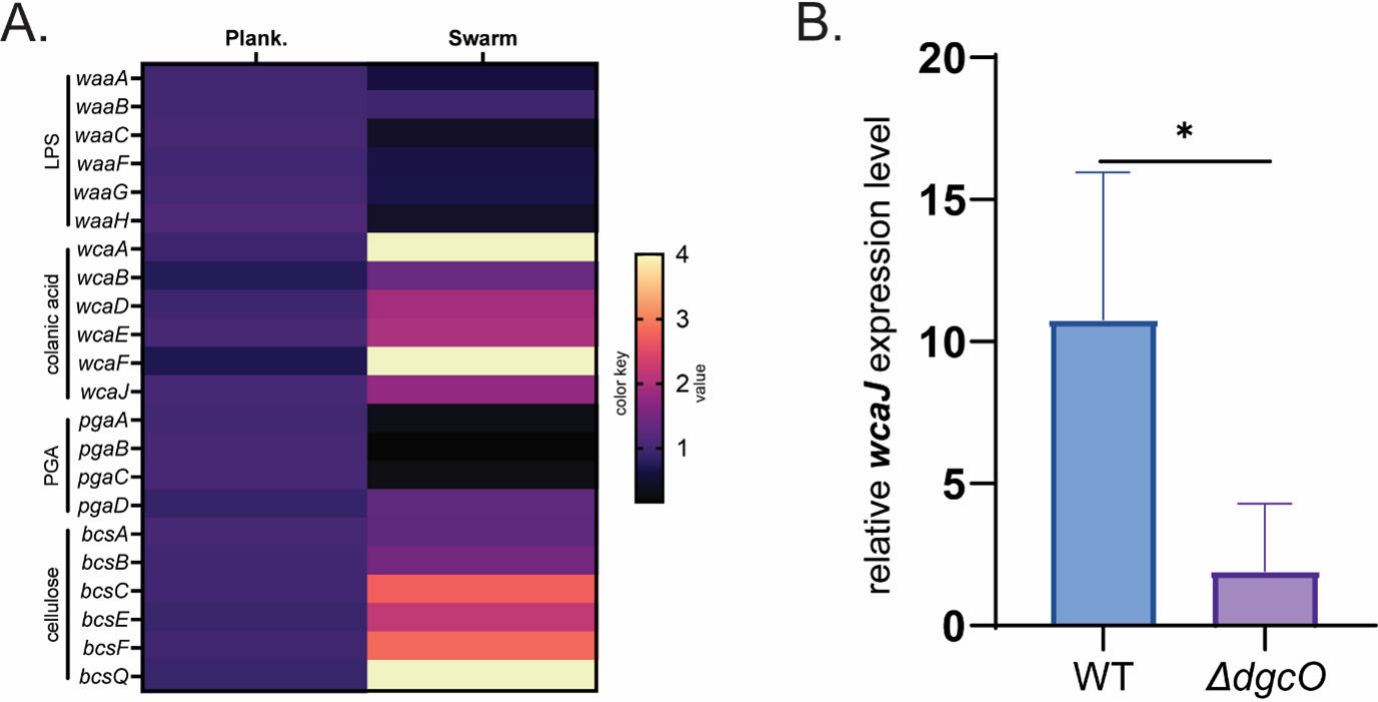
Exopolymer expression profiles during swarming. (**A**) Comparison of fold changes in gene expression of the four exopolymers of *E. coli* (LPS, CA, PGA, and cellulose). RNAseq data collected at 20 h during the time course of swarming were normalized to those from planktonic cultures (*n* = 4). (**B**) Comparison of *wcaJ* expression levels relative to the housekeeping gene *gyrA* in WT and Δ*dgcO,* using qRT-PCR from swarm cells collected at 20 h (see Materials and Methods) (*n* = 3).

Based on our observation that the Δ*dgcO* strain could “move in place” but not venture out (Movie S2), we wondered if the mutant might be defective in the production of polysaccharides that contribute to surface wetting. We therefore examined the RNAseq data for changes in all exopolymers—LPS, CA, PGA, and cellulose. Of these, several genes in the CA (especially *wcaF* and *wcaJ*) and cellulose biosynthetic pathways were seen to be upregulated (Fig. 3A). However, our WT MG1655 strain has a defect in cellulose production (47), so we focused on examining the contribution of CA. Using quantitative reverse transcriptase PCR (qRT-PCR), we confirmed that the expression levels of *wcaJ* were significantly lower in the Δ*dgcO* strain compared to the WT (Fig. 3B), suggesting a possible involvement of this DGC in CA synthesis.

**Fig 4:**
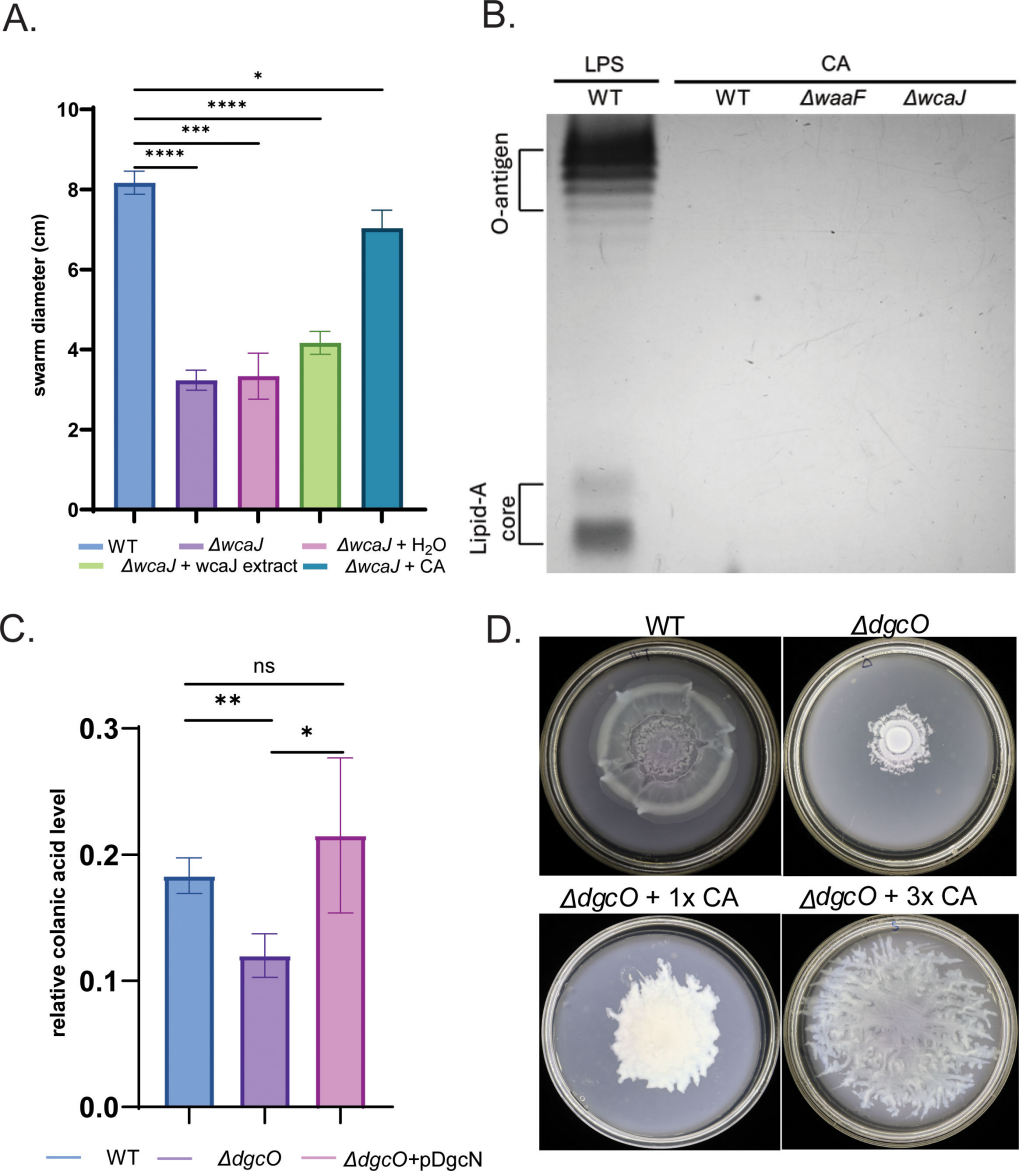
Colanic acid (CA) is required for swarming. (**A**) Swarming diameters at 20 h were compared among WT, Δ*wcaJ* and Δ*wcaJ* supplemented with 100 µL of either water, Δ*wcaJ* extract, or CA extract from Δ*waaF* (*n* = 3). (**B**) LPS extract from WT and CA extracts from Δ*waaF* and Δ*wcaJ* strains were fractionated on SDS-PAGE gel, followed by silver staining (see Materials and Methods). (**C**) Comparison of relative CA levels among WT, Δ*dgcO,* and Δ*dgcO* complemented with pDgcN (*n* = 3). CA levels were measured as described under Materials and Methods. (**D**) Rescue of swarming in Δ*dgcO* by addition of indicated concentrations of CA extract from Δ*waaF*.

To test the role of CA in swarming, we constructed two mutant strains: Δ*wcaJ* and Δ*waaF. wcaJ* is part of a multi-gene operon that controls CA biosynthesis (48); deletion of this gene interferes with CA synthesis but does not affect LPS synthesis. *waaF* belongs to a multi-gene operon controlling LPS biosynthesis (49); deletion of this gene not only produces a defect in the LPS core but also overproduces CA, possibly via the RcsCDB system (49). Δ*wcaJ* was swarming defective (Fig. 4A). Δ*waaF* could not be tested for swarming because flagella biosynthesis is inhibited in this strain (49) via induction of Rcs signaling (50); however, we exploited its CA-overproduction phenotype to extract CA by established protocols (see Materials and Methods), adding it to the non-swarming Δ*wcaJ* directly on the swarm plate to test if external supplementation would rescue the defect (Fig. 4A). Through trial and error, we identified an amount of the extract that fully complemented the Δ*wcaJ* defect (Fig. 4A, +CA). It is important to note that adding the same volume of water as a control was not effective nor was a similar extract made from the Δ*wcaJ* strain (Fig. 4A).

To ensure that our CA extracts were not contaminated with LPS (48, 51), we compared them with LPS extracts from the WT strain using a standard protocol (52). Bands corresponding to LPS in WT extracts were absent in the CA extracts of *wcaJ* and *waaF* mutants (Fig. 4B). We conclude that CA and not LPS complements the *wcaJ* mutant of *E. coli* for swarming.

To determine if the swarming defect of Δ*dgcO* is due to lack of CA production, we first measured their levels in this strain (see Materials and Methods) (Fig. 4C). The Δ*dgcO* strain showed a significant decrease in CA production compared to the WT strain. Additionally, leaky expression from DgcN was sufficient to rescue CA levels in Δ*dgcO,* showing that c-di-GMP is required for maintaining CA production (Fig. 4C). To test if external addition of CA would rescue the *dgcO* defect, we added to it the CA extract from the Δ*waaF* strain. We observed a positive correlation of swarming with the amount of CA added (Fig. 4D), but not with extract from the Δ*wcaJ* strain (Fig. S4A). To ensure that CA addition does not simply promote passive “sliding” (18) was ascertained by adding CA extracts to a Δ*fliC* strain that lacks the external flagellar filament (Fig. S4B). To test if CA production was the primary contributor to swarming, we again used plate conditions where the *dgcO* mutant had a slightly better swarming outcome and combined the mutation with *wcaJ* (Fig. S4C); the effect was not additive. In a related experiment, pDgcN, which complemented the swarming defect of the *dgcO* mutant (Fig. 2A), could not complement that of the *wcaJ* strain (Fig. S4D).

Taken together, the data in this section allow us to conclude that CA is the critical component for surface movement in *E. coli* and that DgcO is primarily responsible for regulating its production. The contribution of DgcM to swarming is likely also through this pathway, given that higher c-di-GMP levels provided by DgcN, expected to stimulate production of other polymers, did not complement the swarming defect in the Δ*wcaJ* strain.

### Addition of glucose rescues Δ*dgcO* swarm defect

**Fig 5:**
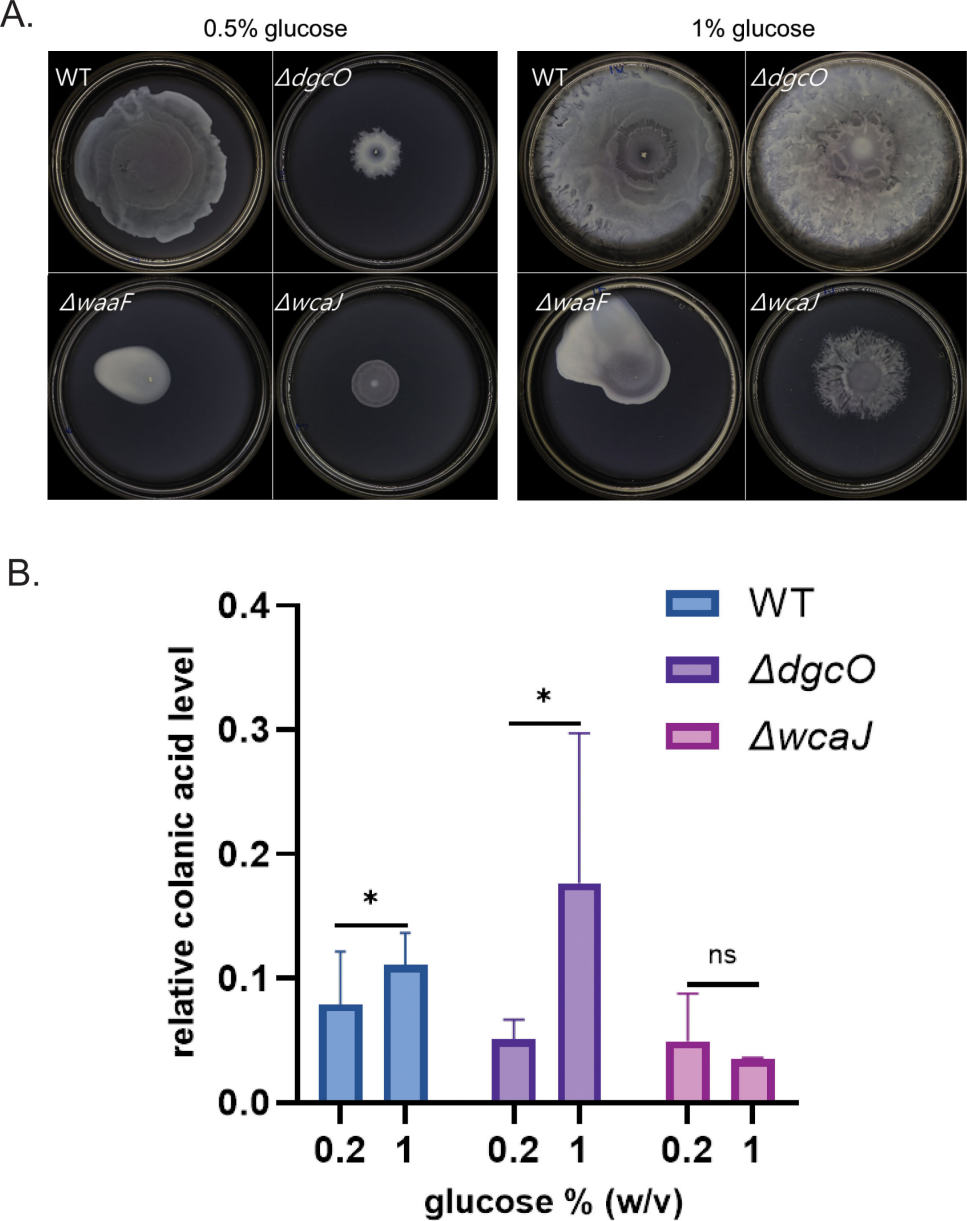
Addition of glucose rescues Δ*dgcO* swarm defect. (**A**) Swarming recorded at 20 h on plates supplied with two different glucose concentrations (0.5% or 1%) and inoculated with indicated strains. (**B**) Comparison of relative CA levels among WT, Δ*dgcO,* and Δ*wcaJ* (*n* = 3).

The availability of glucose has been reported to stimulate the Rcs regulon, which controls CA synthesis (48). *E. coli* requires 0.5% glucose for optimal swarming, and this dependency was traditionally attributed to the need for maintaining the energy-intensive nature of swarming (53, 54). However, examination of WT *E. coli* inoculated on swarm plates with either 0% glucose (no swarming) or 0.5% glucose revealed that cells were similarly motile at the site of inoculation on both plates (Movies S3 and S4). Considering that the addition of CA can rescue Δ*dgcO* cells from the swarm defect (Fig. 4D) and that glucose enhances CA production, we tested whether the addition of glucose could bypass the requirement for c-di-GMP signaling and enable the Δ*dgcO* strain to swarm. Swarming was monitored in four different strains—WT, Δ*dgcO*, Δ*waaF*, and Δ*wcaJ*—on plates supplemented with either 0.5% or 1% glucose (wt/vol). Δ*dgcO* exhibited a full restoration of swarming at 1% glucose (Fig. 5A). In contrast, the Δ*wcaJ* strain showed only a slight increase in swarm diameter, likely due to the absence of a key component of the CA pathway in this mutant. (The lopsided growth evident in the Δ*waaF* strain is due to the highly mucoid nature of excess CA.) These results were further verified by measuring relative CA levels across these plates (Fig. 5B), where addition of glucose increased the CA levels in both WT and Δ*dgcO* but not in the Δ*wcaJ* strain.

We conclude that CA is important for *E. coli* swarming and that c-di-GMP produced by DgcO(/DgcM) is feeding into CA synthesis.

### PGA is semi-essential for swarming; cellulose is not

In *E. coli*, both PGA and cellulose are regulated by c-di-GMP signaling (34, 38, 39, 55–58), with DgcO being implicated in PGA regulation (38). To test the role of PGA in swarming, we constructed two mutant strains, Δ*pgaA* and Δ*pgaB*, that are crucial for PGA production. Neither strain was defective in swimming (Fig. S5A). Under standard conditions for preparing swarm plates, these mutants were not significantly impaired for swarming compared to WT (Fig. S5B, top row). However, when the plates were dried for an extra 30 min (1.5 h), a noticeable decrease in swarming was observed in the mutants compared to the WT. In Δ*pgaA*, this defect was overcome upon complementing *pgaA* from a plasmid (pPgaA), suggesting that PGA plays a niche role for *E. coli* motility on drier surfaces. Addition of glucose did not rescue the defect (data not shown), suggesting that the role of PGA in swarming is distinct from that of CA.

MG1655, the *E. coli* strain used in our study, contains a mutation in the *bcsQ* gene in the cellulose synthesis pathway that introduces an early stop codon, similar to many strains derived from the K-12 lineage (47). This mutation results in reduced cellulose production compared to other *E. coli* strains, such as enteroaggregative (EAEC) ones, that are known to form a robust biofilm (47). To assess the role of cellulose in swarming motility, we reversed the premature stop codon by introducing a single mutation in codon 6 (TAG→TTG, A17T) of *bcsQ* to restore cellulose production as previously reported (47). Indirect measurement of cellulose using crystal violet staining indicated a significant increase in staining (Fig. S5C). However, the reverted strain did no better than the original in swarming, even when plates were dried for an extra 30 minutes (Fig. S5D). From these experiments, we conclude that, among the three polymers *E. coli* secretes, CA plays a major role, PGA plays a secondary role, and cellulose plays no role in swarming motility under our experimental conditions.

### Colanic acid acts as a surfactant

**Fig 6:**
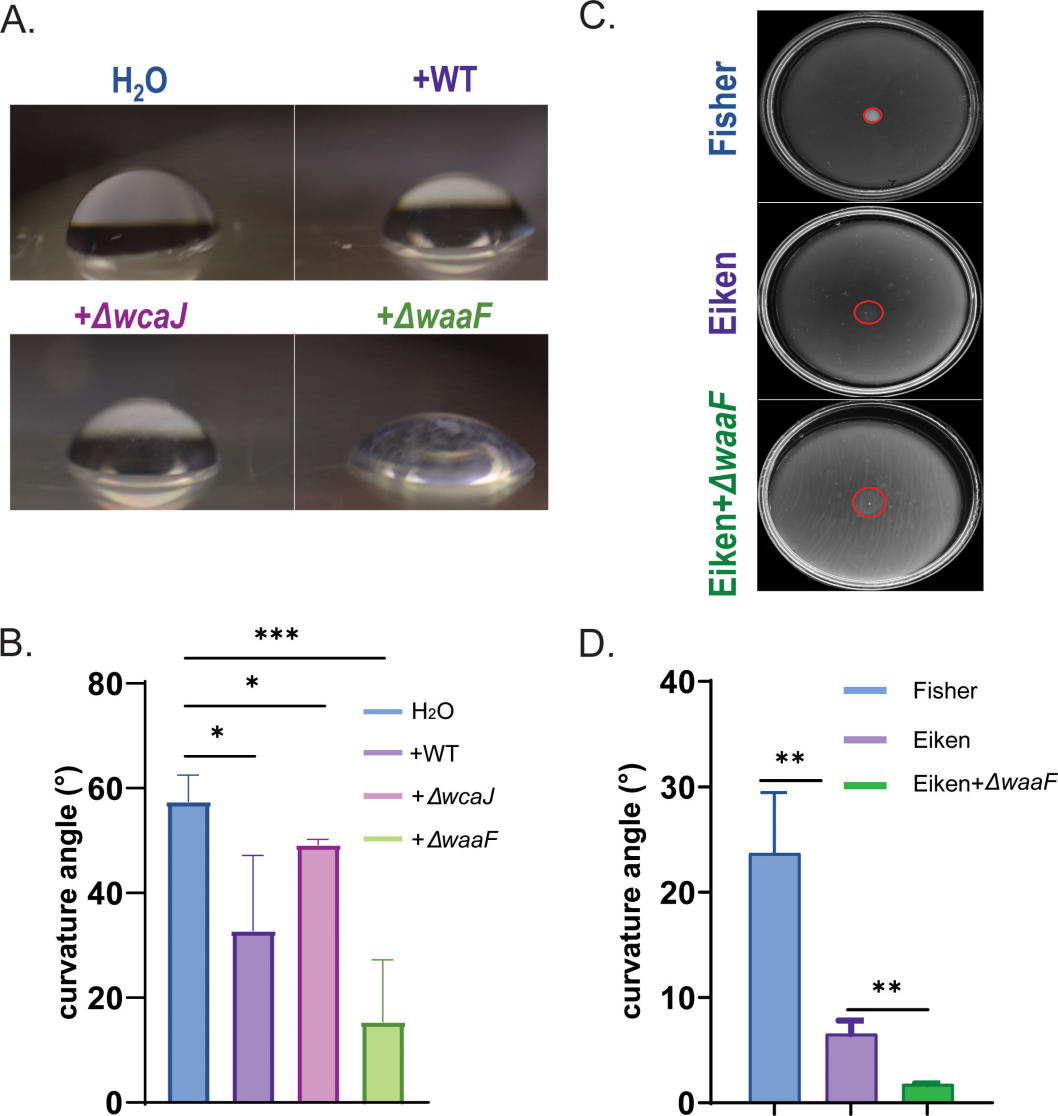
Colanic acid acts as a surfactant. (**A**) A side view of 10 µL water drops or CA extracts in water from indicated strains, deposited on a plastic surface. + indicates CA extract added from indicated strain. (**B**) A summary of contact angles measured directly from the images in panel A (see Materials and Methods). (**C**) Similar to panel A, except water drops were placed directly on swarm media set with either Fisher or Eiken agar (top two plates) or after CA extract from Δ*waaF* was first spread on Eiken agar. (**D**) Contact angles were calculated as described in Materials and Methods.

CA could assist swarming by providing either a wetting or a surfactant function. CA satisfies the former criterion because it is a negatively charged polymer, but we decided to evaluate the latter as well. To do so, we conducted a drop collapse test, a method commonly used to assay surfactants (22), where the curvature angle of the droplet is inversely correlated with its surfactant capacity. CA extracts from WT, Δ*wcaJ*, and Δ*waaF* strains propagated on swarm media were placed on a clean plastic surface, along with water as control (Fig. 6A). The curvature angle of the Δ*wcaJ* extract was comparable to that of water, whereas the Δ*waaF* extract showed a significant decrease (Fig. 6B), indicating that CA indeed has surfactant properties. Next, we compared the curvature angle of water drops placed directly on the surface of swarm media set with either Eiken or Fisher agar (which does not support swarming) (outline marked with red-dotted lines) and on Eiken agar coated with the CA extract (Fig. 6C). Contact angles were calculated by capturing a close-up, top-view image of the drop as previously described (22) (Fig. 6D). The larger the diameter of the drop base is, the smaller the angle of contact is. Fisher agar showed a significantly higher contact angle compared to Eiken agar, indicating that the added water drop experiences lower surface tension on Eiken agar as surmised earlier (24, 59). Eiken agar coated with CA had the lowest contact angle of all. These results suggest that the addition of CA effectively lowers surface tension not only on plastic (Fig. 6A and B) but also on swarm agar plates (Fig. 6C and D). In summary, these data support our conclusion that CA has surfactant properties.

## DISCUSSION

**Fig 7:**
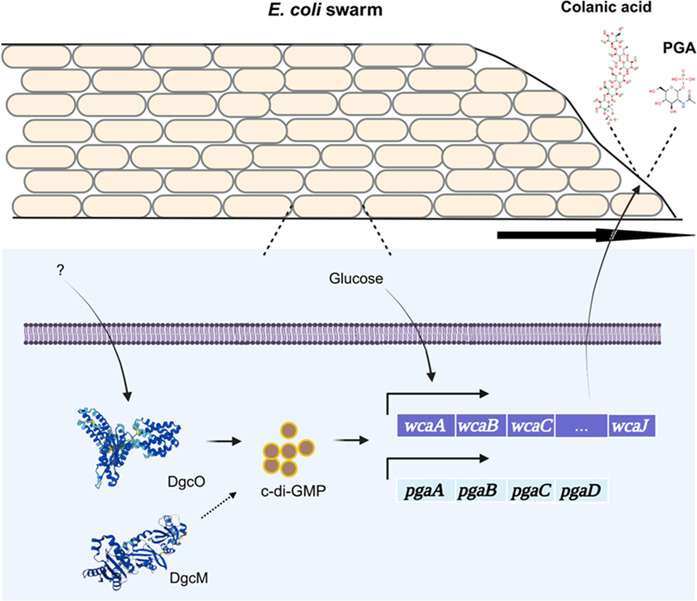
Summary of the role of c-di-GMP in swarming. In response to unknown surface signals (?), *dgcM* and *dgcO* expression is transcriptionally upregulated, leading to an increase in intracellular c-di-GMP. DgcO is a major contributor (solid line), and DgcM is likely a minor contributor (dotted line) to CA synthesis by stimulating *wca* operon transcription. DgcO is also known to promote PGA production by stimulating the *pga* operon transcription. Extracellular CA promotes swarm advancement by lowering surface tension. Both hydrophilic polysaccharides may also contribute to attracting water to the swarm, enabling flagella to work.

c-di-GMP has been thought to signal only one thing, transition to a biofilm. Our work shows that the choice is not binary and that threshold amounts of c-di-GMP signal a habitat conducive to swarming (Fig. 2 and Table 1). Of the three DGCs upregulated during swarming (Fig. 1A), DgcO was found to play a major role and DgcM was found to play a secondary role in facilitating swarming, while no phenotype was evident for DgcJ under our experimental conditions (Fig. 1B and C). We find that DgcO promotes CA synthesis (Fig. 3). CA, which supports swarming when added extracellularly to the *dgcO* mutant (Fig. 4), has surfactant properties that are expected to aid swarming (Fig. 6). While we have not critically examined the DgcM contribution, it likely feeds into the CA pathway (Fig. S2 and S4D). PGA, also known to be controlled by DgcO (38), contributes to swarming on drier surfaces (Fig. S5B). It is possible that DgcJ, which controls production of a different exopolysaccharide (32, 33), enables swarming on other types of surfaces not tested in this study. The main findings of this study are summarized in Fig. 7.

When planktonic bacteria are transferred to a surface, they use a myriad of surface and nutrient cues to change their physiology and adapt (60). Surface cues for initiating swarming in *E. coli* are not known. *E. coli* has multiple DGCs and PDEs (4), all of which contribute to the cellular c-di-GMP pool. Our study shows that signaling specificity can be achieved through environmental cues that filter out suitable DGCs at the transcriptional level. The choice of DgcO and DgcM could reflect the fact that they have autoinhibitory sites (I-sites) (35, 61), which help regulate c-di-GMP levels (62), keeping them low enough to not activate YcgR. A known nutrient cue for swarming in *E. coli* is low intracellular iron (46). In *Pseudomonas aeruginosa,* c-di-GMP levels are regulated by the amount of iron present (63). The connection between iron and c-di-GMP remains to be elucidated in *E. coli*. We note that DgcO possesses a heme domain that interacts with oxygen (37, 64). However, mutations in DgcO reported to interfere with heme binding did not reproduce the Δ*dgcO* swarming phenotype (data not shown).

A particularly gratifying aspect of this study is understanding why the *E. coli* strain we work with needs the special Eiken agar and addition of glucose, a requirement identified 30 years ago (24). We show that Eiken agar has a lower surface tension compared to Fischer agar (which does not support *E. coli* swarming) (Fig. 6C). Addition of glucose contributes further to the lowering of surface tension likely through its known role in stimulating CA synthesis (48). Negatively charged exopolysaccharides have been surmised to function as wetting agents on an agar surface. That CA would also play such a role in *E. coli* is supported by a study that used osmolarity-sensing fluorescent liposomes to calculate that the wetting agent had to be a high molecular weight secreted substance (27). While LPS would fit the bill, our study demonstrated that it is not a major player in our *E. coli* strain (Fig. 4). We note that the robust swarmers *P. mirabilis* and *V. parahaemolyticus* both produce abundant capsular polysaccharides; for *P. mirabilis*, these are required for swarming (65, 66). That such polysaccharides could also serve as surfactants, as shown here for CA, is a new realization.

Finally, while we have established conditions to observe *E. coli* swarming in the laboratory, the composition and texture of surfaces *E. coli* encounters in the environment must vary vastly. The role of DGCs or other enzymes contributing to facilitating collective motion may vary in importance accordingly.

## MATERIALS AND METHODS

### Strains, growth conditions, genetic manipulations, and motility assays

Strains and plasmids used in this study are listed in Table S1. The WT parent strain for *E. coli* was MG1655. Growth media and genetic manipulations have been described earlier (13). Unless stated otherwise, swarm plates were dried at room temperature (RT) for 1 h inside Mystaire MY-PCR prep station laminar flow, prior to inoculation. CA or LPS extracts added were distributed across the surface by gentle shaking with beads (3 mm) (CoilRollers Plating Beads, Novagen Co.).

### Determination of c-di-GMP levels by RFI

This method employs a riboswitch that specifically binds to c-di-GMP, causing a conformational change in the RNA structure that impacts downstream gene expression of red fluorescent protein (RFP). A divergent constitutively active promoter controls cyan fluorescent protein (CFP) expression and normalizes the data for cell number. The relative fluorescence intensity (RFI) of the two readouts at 574 nm (TurboRFP) and 489 nm (AmCyan) is a measure of relative c-GMP levels (41).

All c-di-GMP reporter strains were cultured overnight in a gentamicin-containing medium at 37°C with shaking at 200 rpm before being inoculated onto swim or swarm plates. The plates were incubated for 12 h (swim) or 20 h (swarm) and then stored at 4°C for subsequent experiments. For swarm plate samples, cells were washed off the plates using PBS and resuspended to a final OD_600_ of 0.1. For swim plate samples, the central portion of the agar plate (diameter = 3 cm) was excised, transferred to a 50 mL tube, and washed with PBS. The tube was centrifuged at 1,000 × g for 10 minutes, the supernatant was discarded, and cells from the top of the agar were carefully collected using PBS and adjusted to a final OD_600_ of 0.1. Fluorescence spectra were measured using an RF-5301PC fluorescence spectrophotometer (Shimadzu, Kyoto, Japan). Samples were diluted with water to an OD_600_ of 0.1 before fluorescence measurement.

### Imaging phase contrast view of the edge of a swarm

A phase-contrast microscope (Olympus BX53) equipped with a 40× phase-contrast PH1 objective was used to observe the swarm front. A micro cover glass (18 × 18 mm, VWR) was carefully placed on top of the front, and cell movement was captured using cellSens Imaging Software (Olympus Co.) at a rate of 10 frames per second, with a spatial resolution of 1,004 × 997 pixels and a field of view measuring 120 × 120 µm² for up to 30 s.

### RNA sequencing analysis of swarms

The analysis of *E. coli* swarm cells collected at 2, 4, and 20 h was performed by Marta Perez as part of another project. Swarm cells were harvested at 2, 4, and 20 h after inoculation. Cells were rinsed from the agar plates using a 2:1 mixture of RNAprotect Bacteria Reagent (Qiagen) and PBS and were collected in 1.5 mL test tubes. A total volume of 1 mL was used to rinse all the cells present on each plate. Once collected, the bacterial suspension was vortexed for 5 seconds to mix thoroughly and then incubated at room temperature for 5 minutes. Cells were pelleted by centrifugation at 5,000 × g for 10 minutes, and the supernatant was discarded. Total RNA was isolated from the cell samples treated with RNAprotect Bacteria Reagent using the RNeasy Mini Kit (Qiagen), following the enzymatic lysis and Proteinase K digestion protocol. A maximum of 6 × 10⁸ cells were processed per column. To prevent DNA contamination, we performed on-column DNA digestion using the RNase-free DNase Set (Qiagen), following the manufacturer's instructions. RNA was eluted in 40 µL of RNase-free water and immediately kept cold.

RNA sequencing was outsourced to Novogene. RNA quality was assessed via visualization on agarose gel and determination of RNA concentration and RNA integrity number (RIN) using a Bioanalyzer (Agilent). The RNA quantity used for sequencing was at least 0.5 µg, with a minimum concentration of 10 ng/µL. The RIN values were ≥6 with a smooth baseline. OD_260/280_ and OD_260/230_ ratios were 2.0 or higher. Sequencing was performed on the Illumina NovaSeq 6000 system with a sequencing depth of 6.7 million reads per sample and a read length of 150 bp (paired-end). The expression data were provided as fragments per kilobase of transcript per million mapped reads (FPKM values), calculated by dividing the number of fragments mapped to a gene by the length of that gene in kilobases, followed by normalizing by the total number of mapped fragments in millions (Data set S1).

### Quantitative reverse transcriptase PCR

*E. coli* cells grown on swarm plates (at 20 h) were collected with PBS and resuspended in a final concentration of OD_600_ = 1. RNA was then purified using the Qiagen RNeasy Mini kit (Qiagen Co.) according to the manufacturer's protocol. RNA concentration was determined using NanoDrop One (Thermo Scientific). RNA (1 µg) from each sample was subjected to one-step qPCR using SuperScript III One-Step (Thermo Fisher Scientific), according to the manufacturer's protocol. qRT-PCR reactions were performed in triplicates, and fluorescence detection was performed using QuantStudio 7 Real-Time PCR (Thermo Fisher Scientific). RNA expression was normalized to the level of *gyrA*, a housekeeping gene control. The relative gene expression levels of *wcaJ* were calculated from cycle threshold (CT) values using the 2^−ΔC^ method, where Δ*C* = CT(*wcaJ*) − CT(*gyra*) (67).

### Colanic acid extraction

Colanic acid (CA) was extracted and quantified by modification of the following method (68): 50 µL of prepared extracts was then mixed with 4.5 mL of H_2_SO_4_/H_2_O (6:1 vol/vol) and incubated at 100℃ for 20 min. The mixture was cooled to RT, and absorbances were measured at 396 nm and 427 nm. Cysteine hydrochloride (cys) (100 µl, 1 M) was then added, and the absorbances were measured again at 396 nm and 427 nm. Final CA concentration was measured using the following equation.


$$\left[OD_{396}\;cys\;-OD_{396}\;pre\text{-}cys\right]-\left[OD_{427}\;cys-OD_{427}\;pre\text{-}cys\right]$$

### Extraction and silver staining of LPS

LPS was extracted by hot phenol-water method as described previously with some modifications (51). To remove protein and nucleic acids from cell extracts, we added proteinase K (50 µg/mL) (Roche, Mannheim, Germany), RNase (40 µg/mL) (Roche, Mannheim, Germany), and DNase (20 µg/mL) (Roche, Mannheim, Germany) to the cell extract in the presence of 1 µL/mL 20% MgSO_4_ and incubated them at 37°C overnight. An equal volume of hot (65°C) 90% phenol was added to the mixtures, followed by vigorous shaking at 65°C for 15 min. Suspensions were then cooled, transferred to 1.5 mL polypropylene tubes, and centrifuged at 8,500 × g for 15 min. Phenol phases were re-extracted by 300 µL distilled water. Sodium acetate at 0.5 M final concentration and 10 volumes of 95% ethanol were added to the extracts, and samples were stored at −20°C overnight. After centrifugation, pellets were washed twice with 95% ethanol and resuspended in 120 µL Laemmli sample buffer (33 mM Tris-HCl, pH 6.8, 1% SDS, 13.3% [wt/vol] glycerol, and 0.005% bromophenol blue). Samples were then heated at 100℃ for 20 min. We separated 10 µL of each sample (1 × 10^9^ cells) on 15% SDS polyacrylamide gel with a 5% stacking gel at 100 mA for 1.5 h. Silver and Coomassie blue staining of the gels was performed according to the standard protocols (69).

### Biofilm assay using crystal violet

Biofilm was quantified by using the method in reference 70.

### Contact angle measurement

Water or bacterial culture supernatant drops (10 µL in volume) were placed on the outer side of the bottom part of 100 × 15 mm plastic petri dishes (0875712; Thermo Fisher Scientific). The petri dishes were washed with ethanol. For small angles of contact, where sideview images are not accurate, i.e., <30°, the diameter of each drop was determined by taking a close-up, top-view picture of the drop using a 24.2-megapixel camera (EOS R6 Mark II, Canon) with a 60 mm lens. Pictures were taken 5 min after drop deposition. The angle of contact, θ (°), between drop and surface was determined using a spherical-cap-shaped approximation for small angles (θ < 30°) through θ = 720·*V*π2·*a*3 (where *V* is the volume of the drop [10 µL] and *a* is the radius [mm] of the circle formed by the drop base) as previously described (22).

## Data Availability

RNA sequencing data for *E. coli* genes in each sample, reported in FPKM units, are provided in Data set S1.

## SUPPLEMENTAL MATERIAL

Data set S1 mbio.00916-25-s0001.xlsx RNA sequencing data for *E. coli* genes in each sample.

Supplemental material mbio.00916-25-s0002.docx Supplemental figures and legends for Movies S1 to S4.

Table S1 mbio.00916-25-s0003.docx Strains and plasmids.

Movie S1 mbio.00916-25-s0004.avi Phase contrast view of the edge of a WT swarm.

Movie S2 mbio.00916-25-s0005.avi Phase contrast view of the edge of a Δ*dgcO* swarm.

Movie S3 mbio.00916-25-s0006.avi Movement at the edge of WT *E. coli* swarm without glucose.

Movie S4 mbio.00916-25-s0007.avi Movement at the edge of WT *E. coli* swarm with 0.5% glucose.

ASM does not own the copyrights to Supplemental Material that may be linked to, or accessed through, an article. The authors have granted ASM a non-exclusive, world-wide license to publish the Supplemental Material files. Please contact the corresponding author directly for reuse.
