# Supplementary material for: c-di-GMP is required for swarming in E. coli, producing colanic acid that acts as surfactant
Source: mBio. 2025 May 6;16(6):e00916-25. doi: 10.1128/mbio.00916-25 (PMC12153312; doi:10.1128/mbio.00916-25)
Supplement: Supplemental material — Supplemental figures and legends for Movies S1 to S4. [file mbio.00916-25-s0002.docx]

**Supplemental Figures**


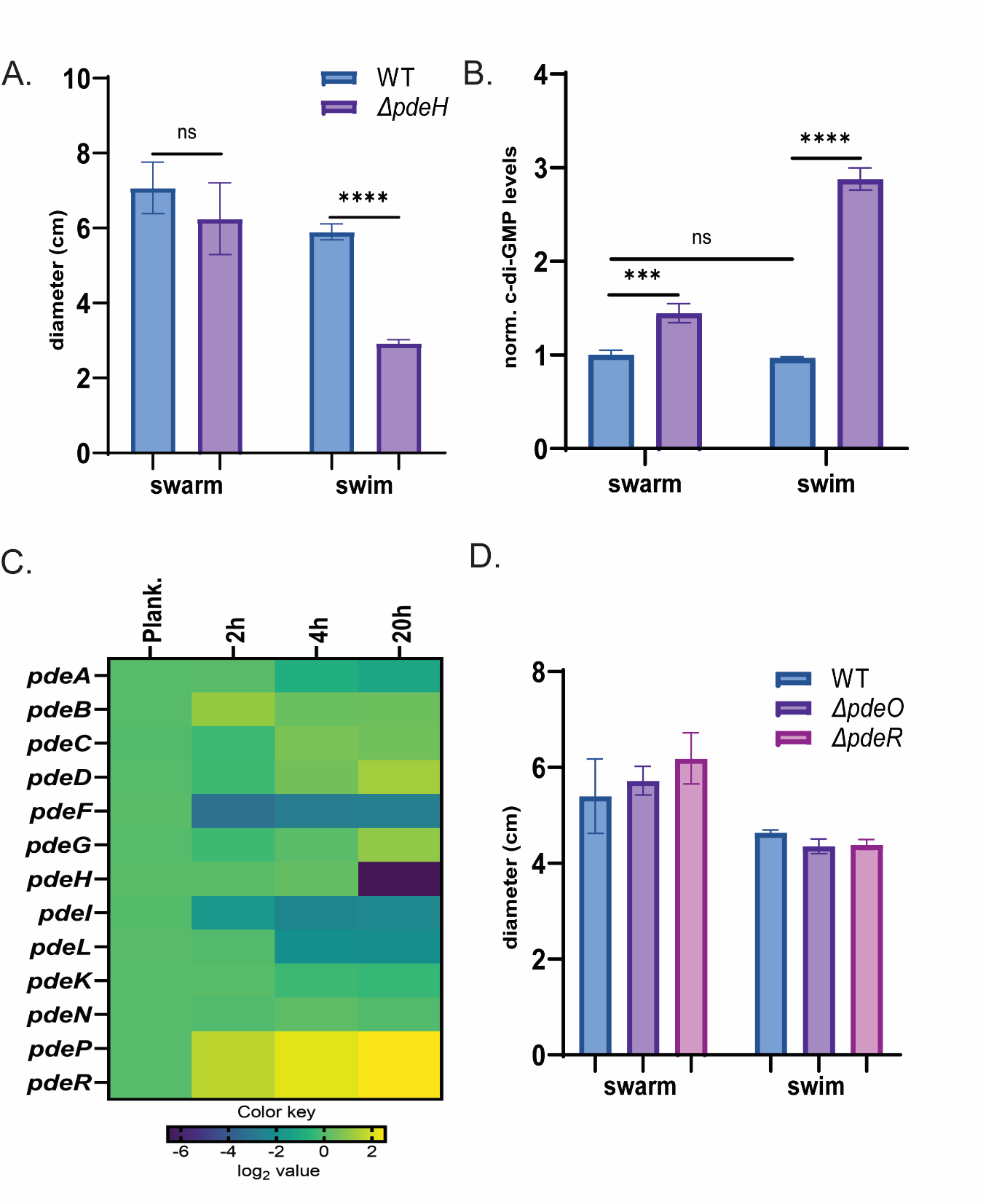


**Figure S1. Differential c-di-GMP levels in swim and swarm cells.** (A) Comparison of motility in swim (0.3% agar) or swarm (0.5% agar) plates of WT (MG1655) and Δ*pdeH* strains. Plates were incubated at 30℃ for 18h (n=3). (B) Measurement of c-di-GMP levels using a riboswitch-based c-di-GMP sensor (see Methods) in cells collected from swim and swarm plates shown in A, normalized to those of WT swim or swarm cells (see Table 1). Color coding of strains as in A. (C) Comparison of log_2_ fold-changes in gene expression of all the *E. coli* PDEs during the time course of WT swarming. RNA-Seq data collected at 2, 4 and 20 h of swarming were normalized to those from planktonic cultures (n=4). (D) Swim and swarm motility comparison of WT, Δ*pdeO* and Δ*pdeR* strains as in A.


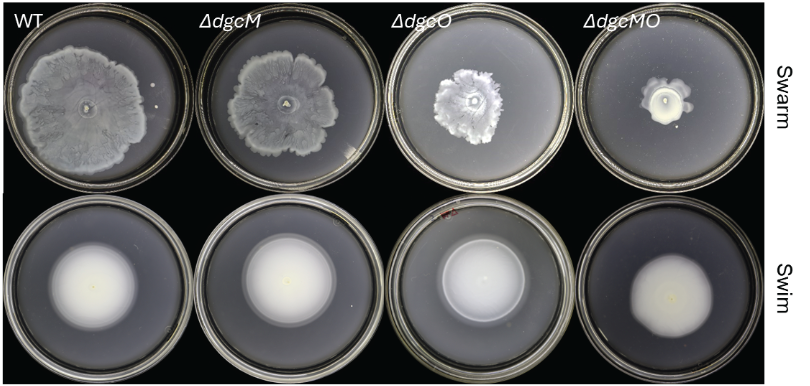


**Figure S2. Comparison of swim/swarm motility in *ΔdgcM, ΔdgcO,* and *ΔdgcMO* strains**. Experimental setup as in Figure 1B, except plates were dried for 50 min instead of an hour and incubated at 30℃ for 18h (n=3).


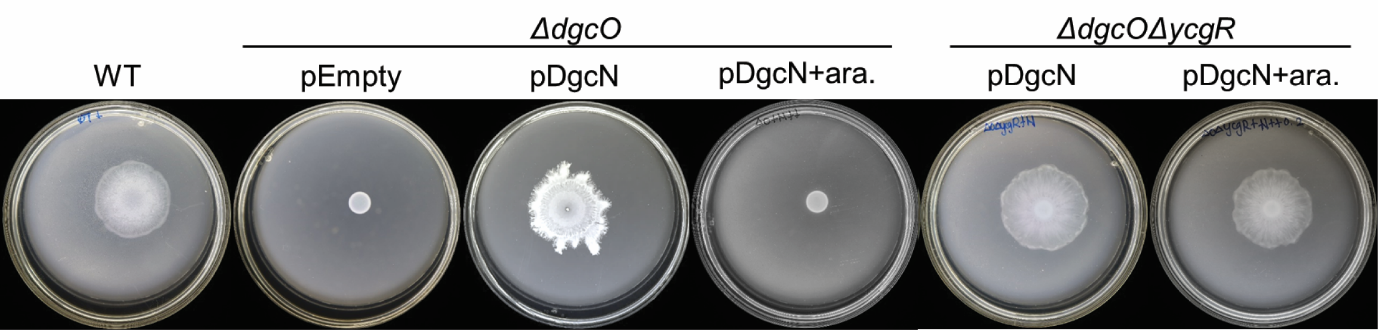


**Figure S3. Swarming inhibition of *ΔdgcO* by pDgcN induction is through YcgR**. Experimental setup as in Figure 2A except also monitored in a *ΔdgcOΔycgR* strain (n=3).


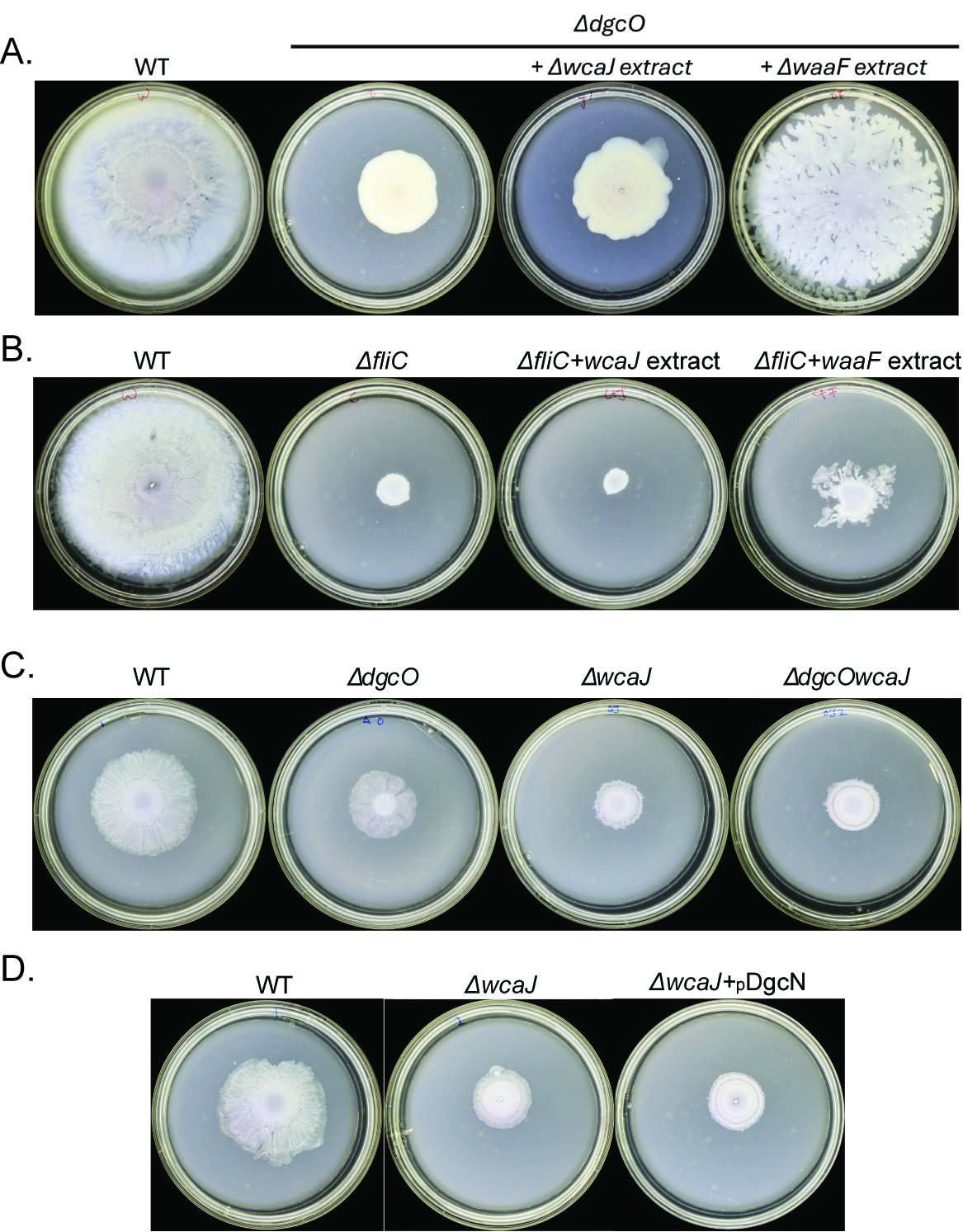


**Figure S4.** **DgcO primarily promotes CA synthesis.** (A) CA extracts from *ΔwcaJ* or *ΔwaaF* strains were added to *ΔdgcO* to observe rescue of swarming. (B) CA extracts from *ΔwcaJ* and *ΔwaaF* strains added to a Δ*fliC* strain to test for growth-fueled passive sliding. (C) Comparison of swarming of indicated single and double deletion mutants. Plates were dried for 50min. and incubated at 30℃ for 18h. (D) As in Fig 2A, except in *ΔwcaJ* background. N=3 in all experiments.


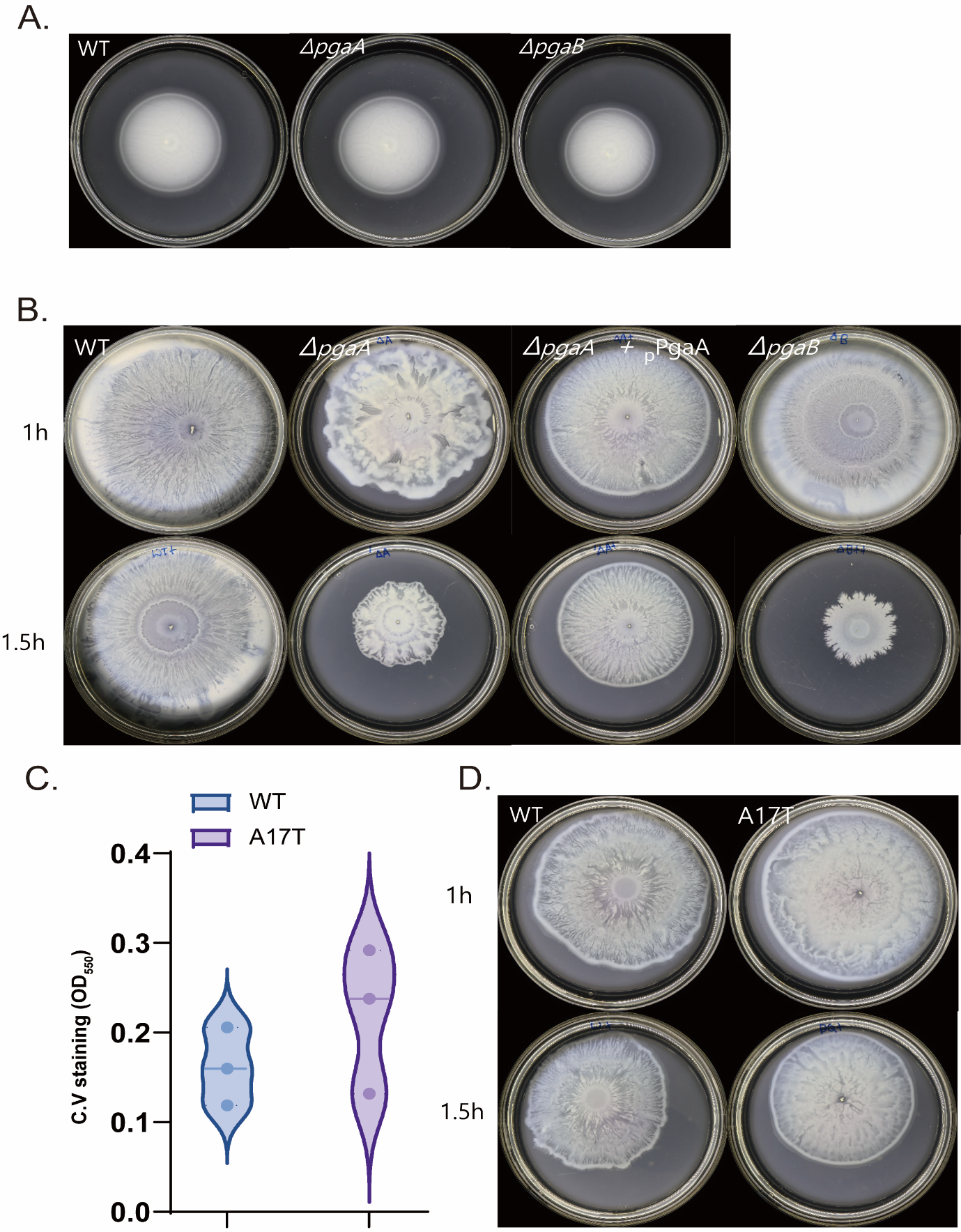


**Figure S5. PGA, but not cellulose, assists swarming motility.** (A-B) Comparison of swimming and swarming of indicated *pga* deletion mutants of MG1655. Swarm plates were incubated at 30℃ for 18h and swim plates at 37℃ for 12h (n=3). Swarm plates were dried under the hood for 1h (standard condition) or 1.5h. (C) Biofilm levels measured levels at OD_550nm_ by crystal violet staining of WT and *bcsQ*(A17T). (D) Swarming in indicated strains. Plates were dried for either 1 or 1.5h before cells were inoculated and incubated at 30℃ for 18h.

**Movie S1. Phase contrast view of the edge** **of a WT swarm**. Cells inoculated on swarm agar (see Methods) were viewed under phase contrast after 4h of incubation at 30ºC, a time when movement is generally first observed. The movie is in real time, at 40x magnification.

**Movie S2. Phase contrast view of the edge** **of a Δ*dgcO* swarm**. Experimental protocol was as described in Movie S1.

**Movies S3. Movement at the edge** **of WT *E. coli* swarm under without added glucose. different glucose concentrations**. Experimental conditions as described for Movie S1, except glucose was not added to the plates.

**Movie S4. Movement at the edge** of **WT *E. coli* swarm with added glucose**. Experimental conditions as described for Movie S1, except plates were supplemented with the standard 0.5% glucose.
