# Supplementary material for: c-di-GMP is required for swarming in E. coli, producing colanic acid that acts as surfactant
Source: mBio. 2025 May 6;16(6):e00916-25. doi: 10.1128/mbio.00916-25 (PMC12153312; doi:10.1128/mbio.00916-25)
Supplement: Table S1 — Strains and plasmids. [file mbio.00916-25-s0003.docx]

**Table S1. Strains and Plasmids.**

| **Strains** | **Genotype or Description** | **Reference** |
| --- | --- | --- |
| MG1655 | K12 wild-type (WT) strain: F^-^, λ^-^, rph-1 | Laboratory Collection |
| JH702 | MG1655 ∆*dgcM* | This work |
| JH703 | MG1655 ∆*dgcO* | This work |
| JH704 | MG1655 ∆*dgcMO* | This work |
| JH1011 | MG1655 ∆*dgcO +* pBAD30_empty | This work |
| JH1012 | MG1655 ∆*dgcO +* pBAD30_DgcO | This work |
| JH1013 | MG1655 ∆*dgcO +* pBAD30_YfIN | This work |
| JH706 | MG1655 ∆*waaF* | This work |
| JH707 | MG1655 ∆*wcaJ* | This work |
| JH711 | MG1655 + ASKA_pdeH | This work |
| JH1422 | MG1655 ∆*dgcM +* pBAD30_empty | This work |
| JH1423 | MG1655 ∆*dgcM +* pBAD30_DgcM | This work |
| JH1408 | MG1655 + cdiGsens | This work |
| JH1409 | MG1655 *ΔdgcM*+ cdiGsens | This work |
| JH1410 | MG1655 *ΔdgcO*+ cdiGsens | This work |
| JH1418 | MG1655 *ΔpdeH*+ cdiGsens | This work |
| JH1105-1114 | MG1655 *ΔdgcO* suppressors | This work |
| JH1419 | MG1655 ASKA_PdeH+cdiG sens | This work |
| JH1201 | MG1655 ∆*dgcO*∆*fhuA* | This work |
| JH1202 | MG1655 ∆*dgcO*∆*stfP* | This work |
| JH1203 | MG1655 ∆*dgcO*∆*yhbX* | This work |
| JH1204 | MG1655 ∆*dgcO*∆*fhuA*+cdiG sens | This work |
| JH1450 | MG1655 *∆dgcO∆wcaJ* | This work |
| JH1451 | MG1655 *∆fliC* | This work |
| JH1452 | MG1655*∆wcaJ +* pBAD30_YfIN | This work |
|  |  |  |
| **Plasmids** | Expressed Proteins |  |
| pKD4 | Kanamycin resistance gene template | [71] |
| pKD46 | λ Red Recombinase | [71] |
| pCP20 | FLP recombinase | [71] |
| pBAD30 | Cloning vector; _P_BAD; Amp^R^ | [72] |
| pBAD30_YfiN | pBAD::YfiN | [72] |
| pBAD30_DgcO | pBAD:;DgcO | This study |
| pBAD30_DgcM | pBAD:;DgcM | This study |
| ASKA_PdeH | T5-*lacO* (pca24N)::PdeH | [73] |
| cdiG sens | pRP0122-P*be*-*amcyan*_*Bc4*_*turborfp* | [41] |
|  |  |  |
|  |  |  |

71. Datsenko KA, Wanner BL. 2000. One-step inactivation of chromosomal genes in Escherichia coli K-12 using PCR products . Proc Natl Acad Sci USA 97:6640–6645. <https://doi.org/10.1073/pnas.120163297>

72. Hwang Y, Harshey RM. 2023. A second role for the second messenger cSecond Role for the Second Messenger Cyclic-di-GMP in E. coli: arresting cell growth by altering metabolic floArresting Cell Growth by Altering Metabolic Flow. MBio 14:e0061923. https://doi.org/10.1128/mbio.00619-23

73. Kitagawa M, Ara T, Arifuzzaman M, Ioka-Nakamichi T, Inamoto E, Toyonaga H, Mori H. 2005. Complete set of ORF clones of Escherichia coli ASKA library (a complete set of E. coli K-12 ORF archive): unique resources for biological research. DNA Res 12:291–299. https://doi.org/10.1093/dnares/dsi012
